# Hypothalamic *Hnscr* regulates glucose balance by mediating central inflammation and insulin signal

**DOI:** 10.1111/cpr.13332

**Published:** 2022-08-30

**Authors:** Ya Liu, Yi‐Fan Guo, Hui Peng, Hai‐yan Zhou, Tian Su, Mi Yang, Qi Guo, Xiao Ye, Yan Huang, Tie‐Jian Jiang

**Affiliations:** ^1^ Department of Endocrinology, Endocrinology Research Center Xiangya Hospital of Central South University Changsha Hunan China; ^2^ National Clinical Research Center for Geriatric Disorders Xiangya Hospital of Central South University Changsha Hunan China

## Abstract

**Objectives:**

Hypothalamic dysfunction leads to glucose metabolic imbalance; however, the mechanisms still need clarification. Our current study was to explore the role of hypothalamic *Hnscr* in glucose metabolism.

**Materials and Methods:**

Using *Hnscr* knockout or htNSC‐specific *Hnscr* overexpression mice, we evaluated the effects of *Hnscr* on glucose metabolism through GTTs, ITTs, serum indicator measurements, etc. Immunofluorescence staining and Western blotting were performed to test inflammation levels and insulin signalling in hypothalamus. Conditioned medium intervene were used to investigate the effects of htNSCs on neuronal cell line. We also detected the glucose metabolism of mice with htNSCs implantation.

**Results:**

*Hnscr* expression decreased in the hypothalamus after high‐fat diet feed. *Hnscr*‐null mice displayed aggravated systematic insulin resistance, while mice with htNSC‐specific *Hnscr* overexpression had the opposite phenotype. Notably, *Hnscr*‐null mice had increased NF‐κB signal in htNSCs, along with enhanced inflammation and damaged insulin signal in neurons located in arcuate nucleus of hypothalamus. The secretions, including sEVs, of *Hnscr*‐deficient htNSCs mediated the detrimental effects on the CNS cell line. Locally implantation with *Hnscr*‐depleted htNSCs disrupted glucose homeostasis.

**Conclusions:**

This study demonstrated that decreased Hnscr in htNSCs led to systematic glucose imbalance through activating NF‐κB signal and dampening insulin signal in hypothalamic neurons.

## Graphical abstract

We discovered the lncRNA *Hnscr* participated in the regulation of high fat diet‐induced insulin resistance. *Hnscr* knockout aggravated diet‐induced insulin resistance. Mechanistically, loss of *Hnscr* reduced trim56 level, leading to NF‐κB activation in htNSCs. *Hnscr*‐deficient htNSCs also contributed to increased inflammation and insulin resistance in neurons finally damaged systematic glucose balance.

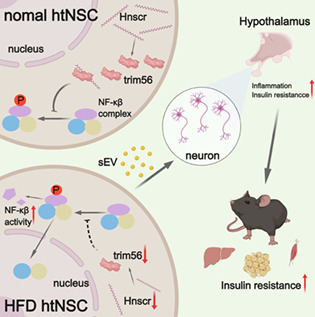

## 1 INTRODUCTION

Over the past few decades, the global prevalence of metabolic syndrome (MS) and type 2 diabetes mellitus (T2DM) increased substantially. Insulin resistance, the major pathological feature of MS and T2DM, is characterized by reduced sensitivity to insulin in various tissues such as the fat, liver, and muscle. The modulation of insulin resistance and glucose metabolism led by peripheral organs were widely documented, and increasing studies have focused on the regulatory role central nervous system in the process.^1^, ^2^, ^3^

Recent studies have shown an atypical form of pro‐inflammatory signalling in the hypothalamus, which is known as ‘hypothalamic microinflammation’.^4^, ^5^ Characterized by the enhanced nuclear factor kappa‐B (NF‐κB) signal pathway, the micro‐inflammatory changes were reported in multiple cell types located in the mediobasal hypothalamus (MBH), including neurons, astrocytes, and htNSCs.^6^, ^7^, ^8^ And enhanced hypothalamic inflammation finally contributes to the development of pre‐diabetes.^9^ HtNSCs were reported to play a leading role in the regulation of the aging process and metabolic disorders.^10^, ^11^ Inflamed htNSCs had impaired neurogenesis capacity, which contributed to the loss of certain neurons,^8^, ^12^ and also had altered secretory patterns,^13^ finally resulting in a metabolic imbalance.

Here, we discovered the lncRNA *Hnscr* participated in the regulation of high‐fat diet‐induced insulin resistance. *Hnscr* was decreased in the hypothalamus after high‐fat diet feeding. Knockout of *Hnscr* aggravated high‐fat diet‐induced glucose clearance and tolerance, contributing to peripheral insulin resistance. Conversely, htNSC‐specific *Hnscr* overexpression ameliorated this phenotype. Mechanically, *Hnscr* stabilized trim56 from degradation, leading to decreased NF‐κB phosphorylation. Through conditioned medium intervention and htNSCs implantation, we found *Hnscr*‐knockout htNSCs further enhanced inflammation and dampened insulin signal in neurons located in the mediobasal hypothalamic region (MBH) through secretions, finally damaged systematic insulin sensitivity.

## 2 RESULTS

### 2.1 *Hnscr* knockout aggravates high fat diet‐induced glucose disorders

**FIGURE 1:**
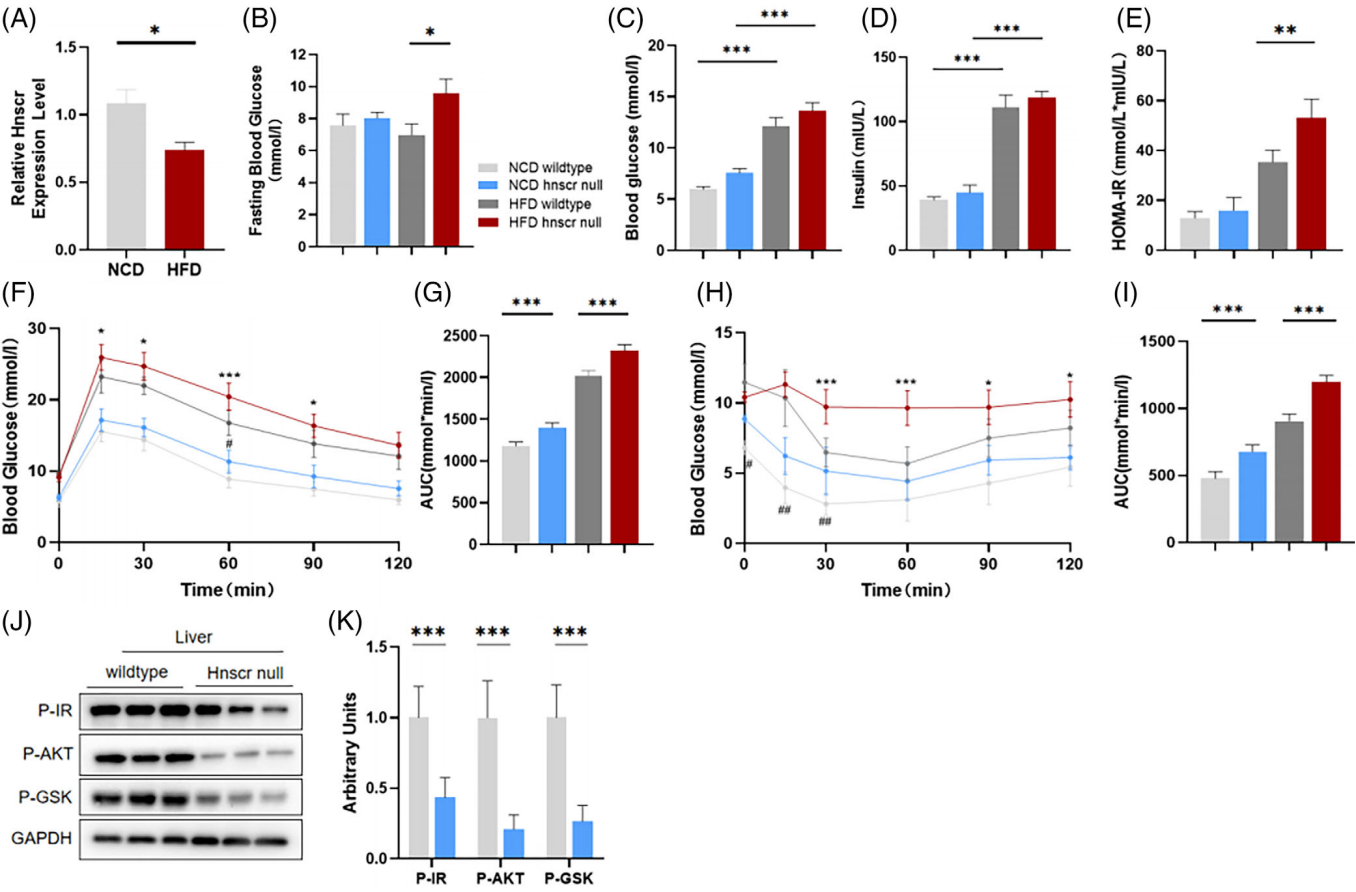
*Hnscr* knockout aggravates high fat diet‐induced glucose disorders. (A) qPCR analysis of *Hnscr* mRNA in hypothalamus of 6‐month old mice fed with 3‐month normal chow diet and high fat diet. 2‐Month old *Hnscr*‐null mice and its age‐matched wildtype littermate were fed with normal chow diet and high fat diet for 3 months. (B, C) Fasting and fed blood glucose levels. (D) Fasting serum insulin levels. (E) Homeostasis model assessment of insulin resistance (HOMA‐IR) index. (F, G) Glucose tolerance tests. (H, I) Insulin tolerance tests. (J–K) Insulin stimulated IR, AKT, and GSK3β phosphorylation in liver. Data are presented as mean ± SEM (*n* = 5–6). Statistical significance was calculated by two‐tailed Student's *t* test or two‐way ANOVA (*p < 0.05, **p < 0.01, ***p < 0.001, #*p* < 0.05, ##*p* < 0.01)

In previous study, we found the pivotal roles of *Hnscr*, a lncRNA specially enriched in htNSCs, in regulating aging process, and aged *Hnscr* null mice had aggravated aging‐related physiological phenotype.^10^ Since hypothalamus is reported as the headquarter of aging development and whole‐body metabolism,^5^ we further inquired whether *Hnscr* participated in modulating glucose metabolism. To this end, we first detected *Hnscr* level in mice with a normal chow diet (NCD) and high‐fat diet (HFD) and found decreased hypothalamic *Hnscr* level after HFD feed (Figure 1A). *Hnscr* null mice and their age‐matched wild‐type mice were challenged with NCD and HFD for 3 months. *Hnscr* depletion increased fasting blood glucose level after HFD feeding (Figure 1B), with no significant difference in random blood glucose compared with their controls (Figure 1C). Long‐term HFD feed elevated circulating insulin level (Figure 1D). And *Hnscr* null mice had higher level of HOMA‐IR index (Figure 1E), indicating dampened insulin sensitivity induced by *Hnscr* depletion. We also conducted glucose tolerance tests (GTTs) and insulin tolerance tests (ITTs) to evaluate their capacity for glucose tolerance and clearance. *Hnscr* null mice had worse performance in both GTTs and ITTs with or without high‐fat diet feed, revealed by higher glucose level after glucose or insulin stimulation (Figure 1F–I). Additionally, the hepatic insulin signalling was also damaged after *Hnscr* knockout, demonstrated by decreased level of phospho‐IR, phospho‐AKT, and phospho‐GSK3β after insulin stimulation (Figure 1J,K). We also detected parameters related to lipid metabolism. *Hnscr* null mice had aggravated liver steatosis, with increased liver weight and greater liver size (Figure S1a,b). HE and Oil Red O staining demonstrated an exacerbation in hepatic lipid accumulation caused by *Hnscr* deficiency (Figure S1c), along with increased hepatic triglyceride (TG) and cholesterol (TC) level (Figure S1d–e). Serum lipid measurements revealed that *Hnscr* null mice had decreased TG level and enhanced serum TC level (Figure S1f–h). Consistently, RT‐qPCR analysis also revealed increases in lipogenesis‐ related genes (Figure S1i,j). Serum ALT, markers of liver injury, was increased in *Hnscr* null mice (Figure S1k). These results indicate that *Hnscr* knockout aggravates systematic insulin resistance and liver steatosis.

### 2.2 Hypothalamic *Hnscr* overexpression attenuates systematic insulin resistance

**FIGURE 2:**
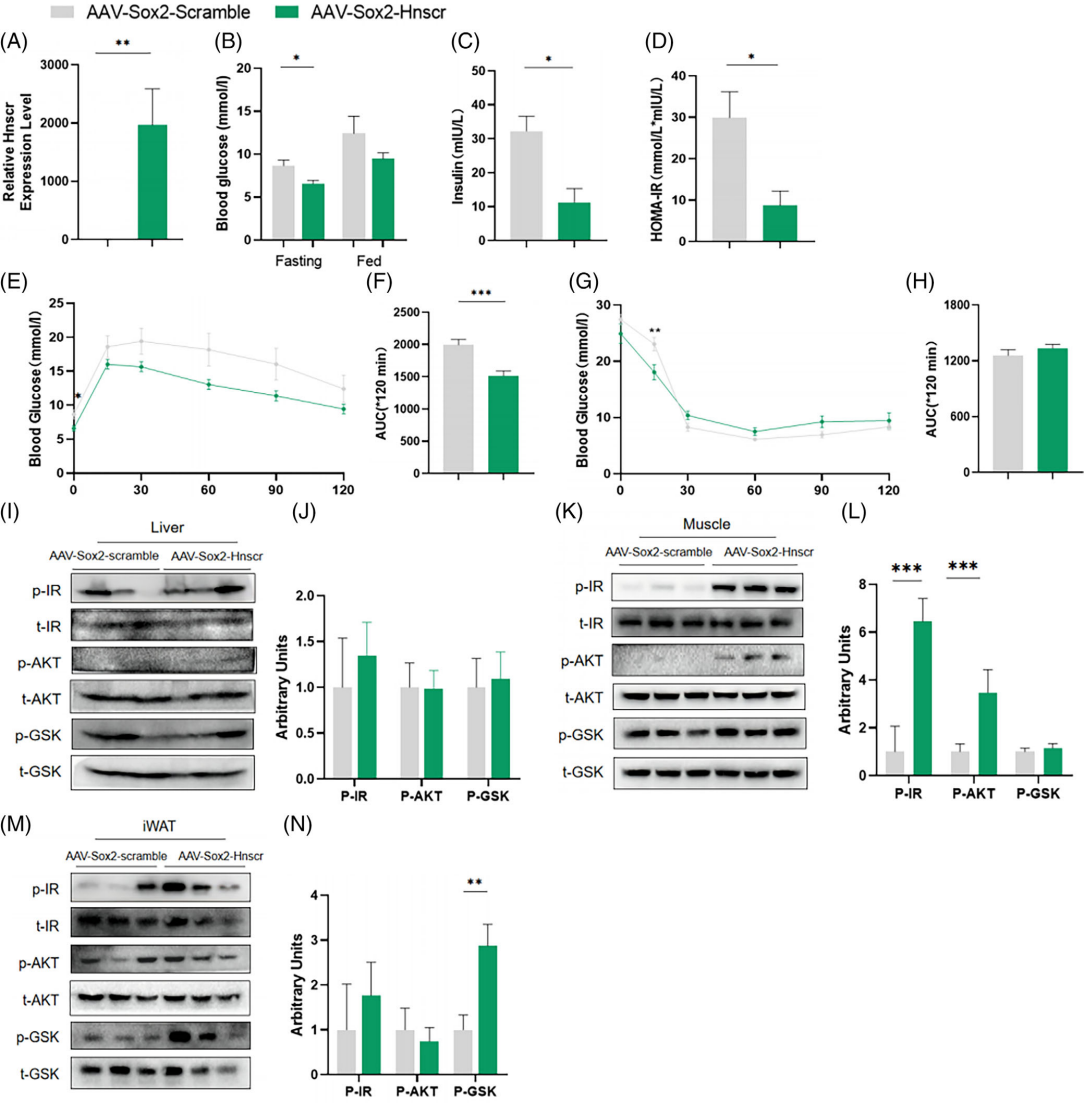
Hypothalamic *Hnscr* overexpression attenuates systematic insulin resistance. AAV‐Sox2‐*Hnscr* and its control AAV‐Sox2‐Scramble were injected into mediobasal hypothalamic (MBH) region of 2‐month old mice, following high‐fat diet‐feed for 3 month. (A) qPCR analysis of *Hnscr* mRNA in hypothalamus 1 month after AAVs injection. (B) Fasting and fed blood glucose levels. (C) Fasting serum insulin levels. (D) HOMA‐IR index. (E–H) Glucose tolerance tests and insulin tolerance tests. (I–N) Insulin stimulated IR, AKT, and GSK3β phosphorylation in liver, muscle, and inguinal white adipose tissue (iWAT). Data are presented as mean ± SEM (*n* = 5–6). Statistical significance was calculated by two‐tailed Student's *t* test (*p < 0.05, **p < 0.01, ***p < 0.001)

To confirm the regulatory effects of hypothalamic *Hnscr* in glucose metabolism, we generated a mice model with htNSC‐specific *Hnscr* overexpression by injecting AAV‐Sox2‐*Hnscr* into the mediobasal hypothalamic region (MBH), and mice injected with AAV‐Sox2‐Scramble were used as controls. Quantitative real‐time reverse transcription PCR (qRT‐PCR) analysis confirmed the highly expressed *Hnscr* in hypothalamus after AAV‐Sox2‐*Hnscr* injection (Figure 2A). And two groups of mice were fed with HFD for 3 months after injection. Interestingly, mice with hypothalamic *Hnscr* overexpression had decreased level of fasting glucose, while no significant difference was in fed blood glucose (Figure 2B). Decreased serum insulin level and improved HOMA‐IR index were also found in mice with AAV‐Sox2‐*Hnscr*, compared with the controls (Figure 2C,D). More importantly, mice with hypothalamic *Hnscr* overexpression had decreased blood glucose level after glucose administration in GTTs, while no significant difference in glucose level of ITTs (Figure 2E–H). *Hnscr* overexpression also contributed to activated insulin signalling, with increased phospho‐IR, phospho‐AKT, and phospho‐GSK3β expression in insulin‐sensitive tissues (Figure 2I–N). No significant differences were found in the liver weight or morphology among these two groups (Figure S2a–c), which prompted us to mainly focus on the altered glucose metabolism led by *Hnscr*. These data demonstrate that *Hnscr* overexpression in htNSCs alleviates high‐fat diet‐induced insulin resistance, indicating the regulatory role of *Hnscr* in glucose metabolism.

### 2.3 *Hnscr* knockdown in htNSCs enhances inflammation in hypothalamus

**FIGURE 3:**
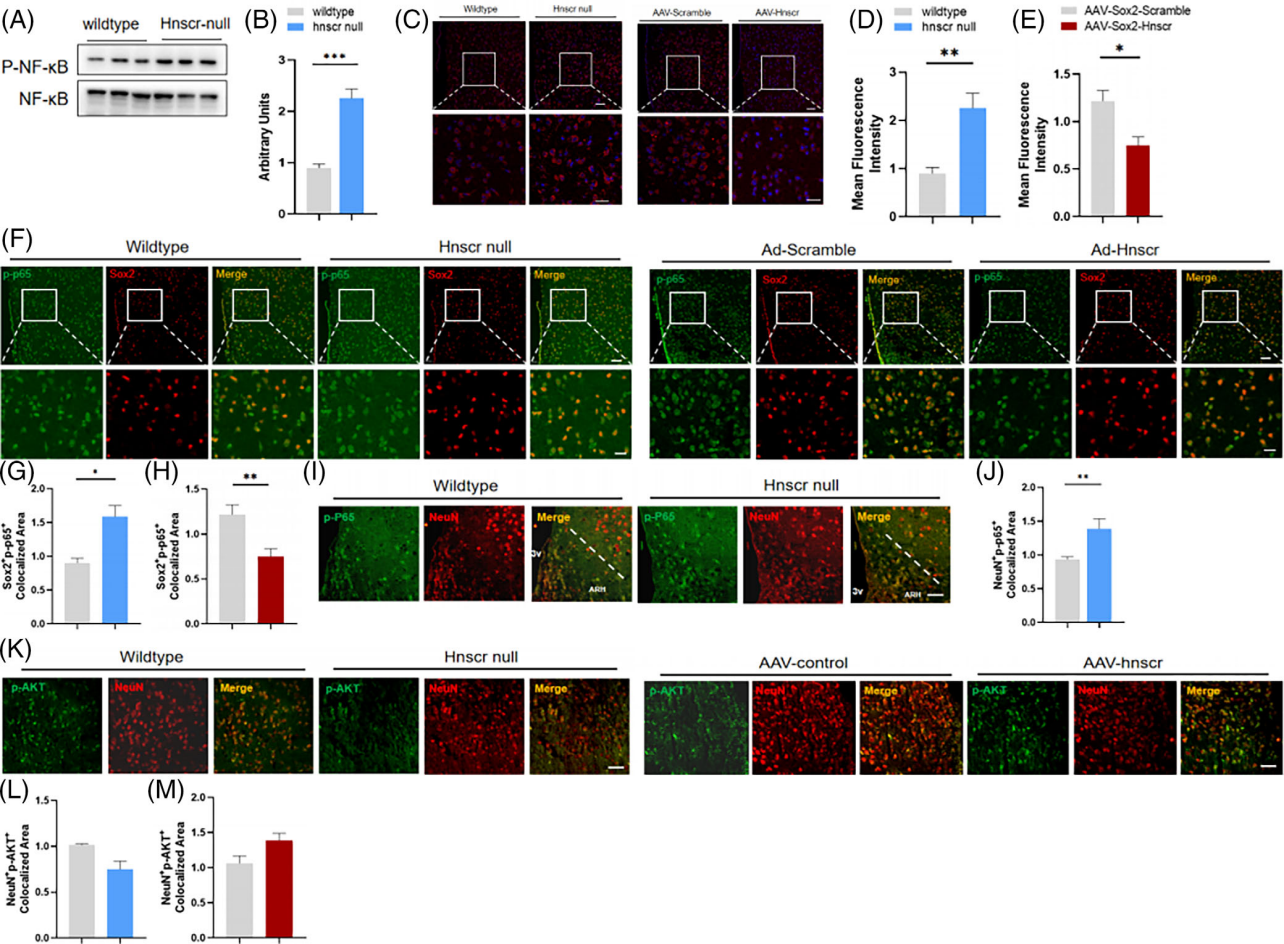
*Hnscr* knockdown in htNSCs enhanced inflammation in hypothalamus. (A, B) Western blot of NF‐κB phosphorylation in hypothalamus of *Hnscr*‐null and wildtype mice fed with high fat diet. (C–E) Immunofluorescence staining for TNFα (red) in ARH. Scale bar: 50 μm. (F–H) Immunofluorescence staining for Sox2 (red), phospho‐NF‐κB (green), and merge (yellow) in ARH. Scale bar in upper line: 100 μm, scale bar in upper line: 50 μm. (I, J) Immunofluorescence staining for NeuN (red), phospho‐NF‐κB (green), and merge (yellow) in ARH. Scale bar: 50 μm. (K, M) Immunofluorescence staining for NeuN (red), phospho‐AKT (green), and merge (yellow) in ARH after insulin stimulation. Scale bar: 50 μm. Data are presented as mean ± SEM (*n* = 3–4). Statistical significance was calculated by two‐tailed Student's *t* test or two‐way ANOVA (*p < 0.05, **p < 0.01, ***p < 0.001)

Increased hypothalamic inflammation contributed to aging process and metabolic dysfunction.^9^ RNA‐sequencing was conducted on htNSCs of *Hnscr* null mice and wild‐type mice in relevant study,^10^ and GO analysis demonstrated the altered inflammatory response pathway based on the DEGs (differentially expressed genes) in *Hnscr*‐null htNSCs. In light of these evidences, we inquired whether *Hnscr* deficiency‐related glucose metabolic disorder was mediated by hypothalamic inflammation. Increased phospho‐NF‐κB was found in the hypothalamus of *Hnscr*‐null mice with HFD, compared with controls (Figure 3A,B). Correspondingly, *Hnscr* knockout also led to enhanced TNFα expression in the arcuate nucleus area (Figure 3C,D). Immunofluorescence demonstrated increased phospho‐NF‐κB level in sox2^+^htNSCs of *Hnscr*‐null mice (Figure 3F–G). Interestingly, elevated phospho‐NF‐κB were also found in neurons located in arcuate nucleus of hypothalamus (ARH) (Figure 3I,J). In contrast with the enhanced inflammation, mice with htNSC‐specific *Hnscr* overexpression had the opposite phenotype, with reduced hypothalamic TNFα level (Figure 3C,E) and decreased phospho‐NF‐κB expression in Sox2^+^htNSCs (Figure 3F,H).

Besides inflammation, the hypothalamic action of insulin signalling also affects glucose homeostasis.^23^ We further investigated whether insulin signal in hypothalamus were affected by *Hnscr*. And immunofluorescence showed the reduced level of phospho‐AKT in neurons in *Hnscr*‐null mice (Figure 3K,L). And opposite results were found in neurons after *Hnscr* overexpression in htNSCs (Figure 3K–M).

These findings indicate that *Hnscr* expression in htNSCs affects inflammatory status not only in htNSCs per se, but also in neurons located in ARH, and altered insulin signal activation in neurons of ARH.

### 2.4 *Hnscr* attenuates NF‐κB activation by stabilizing Trim56

**FIGURE 4:**
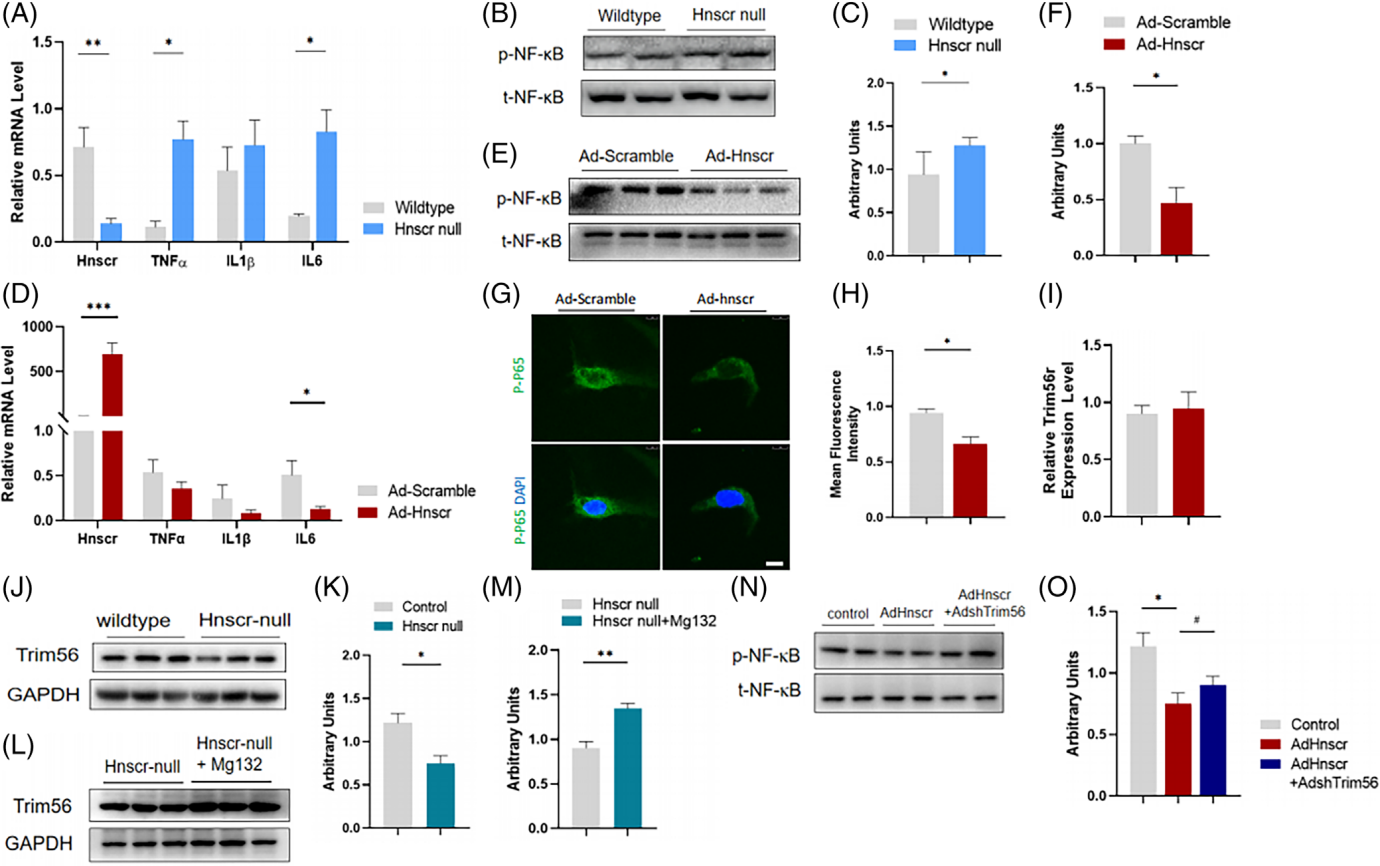
*Hnscr* attenuates NF‐kB activation by stabilizing Trim56 HtNSCs were isolated from *Hnscr*‐null and wildtype mice. (A) qPCR analysis of *Hnscr* and inflammatory indicators including IL‐1β, IL‐6 and TNFα. (B, C) Western blot of NF‐κB phosphorylation with 20 ng/ml TNFα stimulation. 293T cell line was treated with Ad‐*Hnscr* and Ad‐control. (D) qPCR analysis of *Hnscr* and inflammatory indicators. (E, F) Western blot of NF‐κB phosphorylation with 20 ng/ml TNFα stimulation. (G, H) Immunofluorescence of phospho‐NF‐κB in 293T treated with Ad‐*Hnscr* and its controls. Scale bar: 10 μm. (I–K) qPCR and Western blot of trim56 in htNSCs from *Hnscr*‐null and wildtype mice. (L, M) Western blot of trim56 in htNSCs from *Hnscr*‐null mice after Mg132 treatment. (N, O) Western blot of NF‐κB phosphorylation in Ad‐*Hnscr*‐treated 293T cell which transfected with or without Ad‐shTrim56, and cells with Ad‐Control were used as the control. Data are presented as mean ± SEM (*n* = 5–6). Statistical significance was calculated by two‐tailed Student's *t* test (*p < 0.05, **p < 0.01, ***p < 0.001)

To investigate the underlying mechanism, we isolated htNSCs from *Hnscr* null mice and wild‐type littermates. Consistent with previous findings, htNSCs with *Hnscr* knockout had increased inflammatory cytokines expression, including IL‐1β, IL‐6, and TNFα, revealed by RT‐qPCR (Figure 4A). Enhanced NF‐κB signal pathway, revealed by increased phospho‐NF‐κB were found in *Hnscr*‐depleted htNSCs after TNFα stimulation (Figure 4B,C). We further treated 293T cell line with Ad‐*Hnscr* and found decreased IL‐6 level in mRNA levels (Figure 4D). Consistently, increased *Hnscr* level led to a reduced level of NF‐κB phosphorylation, revealed by blotting and immunofluorescence (Figure 4E–H). Based on the negative regulation of *Hnscr* on NF‐κB activation, we further analysed the RNA pull‐down results performed in the previous study,^10^ to inquire about the binding protein of *Hnscr*. An E3 ubiquitin ligase, interferon‐inducible tripartite‐motif (TRIM) 56 (Trim56) aroused our attention. Members of the TRIM protein are reported as important regulators of immunity and inflammation, and some of them were implicated in NF‐κB activation.^14^, ^15^, ^16^, ^17^ Reduced trim56 expression at the protein level was found after *Hnscr* knockout, rather than mRNA level (Figure 4I–K). Meanwhile, the suppressive effects could be abolished by protease inhibitors treatment (Figure 4L,M), suggesting that *Hnscr* protected Trim56 from proteasome degradation. We further investigated whether trim56 mediated the effects of *Hnscr* on NF‐κB activation, and transfected both Ad‐*Hnscr* and Ad‐shTrim56 into 293 T cell line. Interestingly, decreased phospho‐NF‐κB was partly reversed by trim56 ablation (Figure 4N,O). Hence, reduced *Hnscr* contribute to NF‐κB activation, and this effect can be mediated by decreased trim56 expression.

### 2.5 Secretions of *Hnscr*‐null htNSCs lead to inflammation and insulin resistance in neurons

**FIGURE 5:**
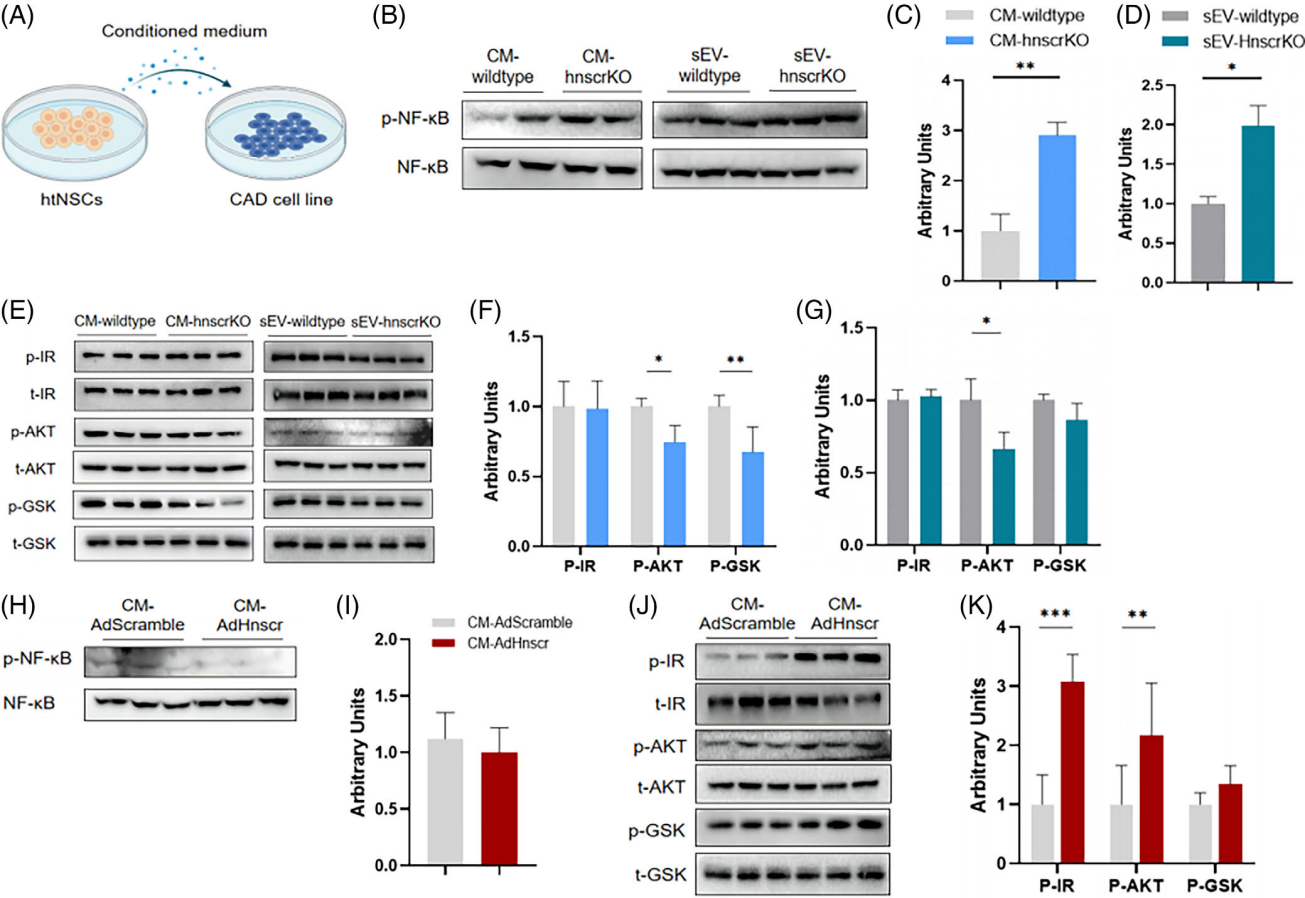
Secretions of *Hnscr*‐htNSCs lead to inflammation and insulin resistance in neurons. (A) Scheme of conditioned medium treatment. AD cell line were treated with conditioned medium of wildtype‐htNSCs (CM‐wildtype) and *Hnscr* null‐htNSCs (CM‐HnscrKO), also sEVs from CM‐wildtype and CM‐HnscrKO for 48 h, followed by detecting insulin signal and NF‐κB phosphorylation. (B–D) NF‐κB phosphorylation after CM‐HnscrKO and CMHnscrKO‐derived sEV treatment. (E–G) Insulin stimulated IR, AKT, and GSK3β phosphorylation after CM‐HnscrKO and CMHnscrKO‐derived sEV treatment. HtNSCs were infected with Ad‐*Hnscr* and Ad‐control, their conditioned medium was extracted, further adding to medium of CAD cell line at ratio of 3:1. (H, I) NF‐κB phosphorylation after CM‐AdControl and CM‐AdHnscr treatment. (J, K) Insulin stimulated IR, AKT, and GSK3β phosphorylation after CM‐AdControl and CM‐AdHnscr treatment. Statistical significance was calculated by two‐tailed Student's *t* test (*p < 0.05, **p < 0.01, ***p < 0.001)

HtNSCs can exert their effects through endocrine ability.^18^ Based on these findings, we hypothesized it was the secretion of htNSCs that led to enhanced inflammation and dampened insulin signal in neurons. Therefore, we extracted the conditioned medium from htNSCs of *Hnscr* null mice (CM‐*Hnscr*KO) and treated the CAD, a CNS cell line (Figure 5A). Consistent with in vivo experiments, 72 h after CM‐*Hnscr*KO treatment, CAD had increased NF‐κB activation (Figure 5B,C). Besides, blunted insulin signal was also found after CM‐*Hnscr*KO administration (Figure 5E,F), as shown by decreased levels of phospho‐AKT and phospho‐GSK. Besides, we further investigated the effects of *Hnscr*‐overexpressed htNSCs, and treated CAD cell line with secretions of htNSCs transfected Ad‐*Hnscr*. Notably, conditioned medium from *Hnscr*‐overexpressed htNSCs (CM‐*Hnscr*OE) led to decreased NF‐κB phosphorylation (Figure 5H,I) and improved insulin signalling (Figure 5J,K). Zhang et al. reported that htNSCs affected hypothalamic inflammation via small extracellular vesicles (sEVs) secretion.^18^ We further inquired whether the changed neuronal phenotype was mediated by htNSC‐derived sEVs. Accordingly, sEVs were isolated from CM‐*Hnscr*KO and CM‐wildtype, subsequently treating CAD cell line. Interestingly, sEVs treatment also enhanced phospho‐NF‐κB expression and partly dampened insulin signalling in recipient cells, which exerted similar effects as the conditioned medium of CM‐*Hnscr*KO (Figure 5B,D–F). To summarize, the secretions of *Hnscr*‐depleted htNSCs aggravated inflammatory response and damaged insulin signalling in recipient neurons, and htNSC‐derived sEVs might be implicated in the process.

### 2.6 *Hnscr*‐null htNSCs implantation impairs systematic insulin sensitivity

**FIGURE 6:**
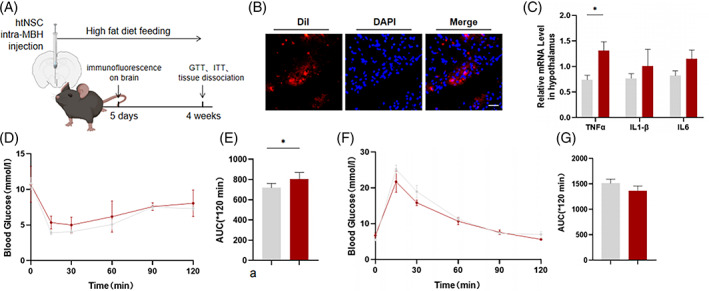
*Hnscr*‐null htNSCs implantation impaired systematic insulin sensitivity. (A) Scheme of *Hnscr*‐null htNSCs implantation. HtNSCs from *Hnscr*‐null and wildtype mice were injected into MBH of recipient mice, followed by high‐fat diet‐feed. Five days after injection, hypothalamus were isolated to detect survival of implanted htNSCs and inflammatory levels. One month later, GTTs and ITTs were conducted. (B) Immunofluorescence of Dil‐labelled htNSCs in MBH. Scale bar: 50 μm. (C) qPCR analysis of TNFα, IL‐1β and IL‐6 in hypothalamus of mice injected with *Hnscr*‐null htNSCs and wildtype htNSCs. (D–G) Glucose tolerance tests and insulin tolerance tests of mice with *Hnscr*‐null htNSCs implantation. Statistical significance was calculated by two‐tailed Student's *t* test (*p < 0.05)

To elucidate the effects of htNSCs in systematic glucose metabolism, we further implanted *Hnscr‐*null htNSCs and control htNSCs into MBH of adult wild‐type mice (Figure 6A). Exogenous htNSCs survived in the MBH area of recipient mice 5 days after injection (Figure 6B). In line with previous findings, *Hnscr*‐null htNSCs implantation led to increased TNFα level in the hypothalamus (Figure 6C), indicating the pro‐inflammatory effects of *Hnscr*‐null htNSCs. After intra‐MBH injection, mice were fed with a one‐month high‐fat diet to investigate whether *Hnscr*‐depleted htNSCs affected the early stage of high‐fat diet‐induced glucose disorder. Mice with *Hnscr*‐depleted htNSCs implantation had dampened ability of glucose clearance, as revealed by ITTs (Figure 6D,E), while no significant difference was found in the GTTs (Figure 6F,G). Taken together, local supplement of *Hnscr*‐depleted htNSCs aggravated central inflammation, and partly exacerbating the high‐fat diet‐induced glucose disorder.

## 3 DISCUSSION

In our study, we found the regulatory role of *Hnscr*, a htNSC‐ abundant lncRNA, in glucose homeostasis. *Hnscr*‐knockout mice had aggravated systematic insulin resistance after HFD feed, while htNSC‐specific *Hnscr* overexpression revealed the opposite phenotype. Further investigation revealed that *Hnscr* depletion in htNSCs enhanced NF‐κB activation and altered secretory pattern, therefore leading to aggravated inflammation and decreased insulin signal in neurons located in the arcuate nucleus of the hypothalamus (ARH). And *Hnscr*‐deficient htNSCs implantation aggravated whole‐body insulin resistance.

HtNSCs are a group of neural stem cells capable of self‐renew and differentiation, which locate in the mediobasal hypothalamus and the third ventricle wall.^5^ HtNSCs are implicated in the regulation of glucose metabolism. IKKβ/NF‐κB activation in htNSCs impaired their multi‐directional differentiating capacity,^8^, ^19^, ^20^ and contributes to decreased POMC neurons, further aggravating obesity and pre‐diabetes phenotype.^8^ HtNSCs also exert their function through the secretion function. Zhang et al. reported that altered miRNA secretory patterns from inflammatory htNSCs accelerated the aging process.^18^ And decreased parathymosin secretion from htNSCs led to enhanced senescence in neighbouring neurons.^21^ In our study, we found that *Hnscr* deficiency in htNSCs increased the vulnerability of NF‐κB activation after TNFα exposure, and vice versa. Besides htNSCs, we also noticed that *Hnscr* ablation enhanced inflammation in ARH neurons, indicating the possible link between htNSCs and neurons. Further study in vitro demonstrated the secretions from htNSCs mediated the effects. Among the secretions, isolated sEVs can also enhance inflammation and insulin resistance in recipient cells, which suggested the sEVs‐packaged contents might be involved in the process. Still, whether and how htNSC‐derived sEVs functioned on neurons need further investigation.

Since hypothalamic insulin signal was reported to be implicated in the regulation of obesity and insulin resistance,^22^ we also investigated the neuronal insulin signalling and found the reduced phospho‐AKT level in neurons after *Hnscr* knockout in htNSCs. Two main populations of neurons, POMC and AgRP, express leptin receptor and insulin receptor,^23^, ^24^ and insulin action in these neurons affected systematic insulin resistance.^25^ Inhibition of AKT phospholation in POMC neurons induced age‐ and diet‐related insulin resistance,^26^, ^27^, ^28^ while, increasing PI3K activity, the upstream of AKT, in POMC neurons improved insulin and glycemic responses.^29^ Thus, we would investigate the implicated neurons in following studies.

In our study, we started from the findings that dampened systematic insulin sensitivity were induced by global *Hnscr* knockout. Inspired by hepatic insulin resistance in *Hnscr*‐null mice, we also constructed transgenic mice with liver‐specific *Hnscr* overexpression to inquire whether *Hnscr* expression in hepatocytes mediated the effects. Unfortunately, hepatic *Hnscr* overexpression failed to alter peripheral insulin sensitivity or liver steatosis after HFD feed (Figure S3). Thus, we turned our attention towards htNSCs, since *Hnscr* were enriched in htNSCs in normal condition and the hypothalamus functioned as the headquarter of whole‐body metabolism. Our study emphasized the importance of maintaining htNSCs homeostasis in glucose metabolism and revealed the crosstalk between htNSCs and neurons.

## 4 METHOD

### 4.1 Animals

8‐Week‐old male C57BL/6J mice were purchased from Hunan SJA Laboratory Animal Company. Male *Hnscr*‐null mice were generated as previously described.^10^ All mice were kept in C57BL/6J background, in SPF class at the Experimental Animal Research Center of Central South University, with a constant temperature of 22–24°C, constant humidity of 60%–75%, 12‐h dark/light cycle, 4–5 mice per cage, free access to feed and water, and regular change of clean bedding. For high fat‐diet (HFD) treatment (60% fat, D12492; Research Diets), mice were fed for 3 months at 8‐week‐old. All animal care protocols and experiments were reviewed and approved by the Animal Care and Use Committee of the Laboratory Animal Research Center, Xiangya School of Medicine, Central South University, and this study complied with all relevant ethical regulations regarding animal research.

### 4.2 Cells

HtNSCs were isolated according to our protocol described previously.^10^ Briefly, the hypothalamic tissues of mice were isolated, then the tissues were shredded and digested with 5 ml of TrypLE™ Express enzyme (Gibco) at 37°C for 30 min. One millilitre pre‐cooled neural cell complete medium (Neurobasal medium (Gibco) + 2% B27 (Invitrogen) + 20 ng/ml EGF (Peprotech) + 20 ng/ml bFGF (Peprotech)) were added to digested tissue to terminate digestion. After centrifugation, precipitates were collected and resuspended in a medium, passed through 100‐μm and 40‐μm cell sieves to separate them into single‐cell suspensions. Single‐cell suspension was inoculated in ultralow‐adhesion 6‐well plates at the density of 10^6^ cells per well. The medium was changed every 3 days. Passed through the generations once in about 2 weeks. Experiments were performed with htNSCs that had been passaged to 3–7 generations.

HEK293T was purchased from Procell Co (China). Cells were cultured in DMEM (Gibco) with 10% fetal bovine serum (Gibco) and 1% penicillin–streptomycin (Procell). CAD cell line were cultured in 10% DMEM/F‐12 (Gibco) with 10% FBS and 1% PS. All cells were cultured at 37°C, 5% CO_2_, and saturated humidity.

### 4.3 Adenovirus infection and TNFα intervention

Adenovirus control (Ad‐Scramble) and Adenovirus‐hnscr (Ad‐*Hnscr*) were generated from Obio Co (Shanghai, China). HtNSCs and 293 T cell line were treated with adenovirus at an MOI of 7 for 2–3 days. Cells were stimulated with TNF‐α at a final concentration of 20 ng/ml for 30 min and collected for further qRT‐PCR and Western blot analysis.

### 4.4 Conditional medium intervention

For conditional medium intervention, the conditioned medium was harvested from htNSCs of wild‐type or *Hnscr*‐null mice, and filtered through 0.22 μm ultrafiltration filters. CAD cells were seeded in 24‐well plates at the density of 2 × 10^6^ cells per well 1 day before the intervention, and treated with a mixed conditioned medium (CM: DMEM/F‐12 medium = 3:1) for 48 h, followed by detection of insulin signal and inflammatory level.

### 4.5 In vitro insulin signalling assay

After the intervention, cells were treated with insulin (Sigma) at a final concentration of 100 nmol/L for 20 min. After washing with sterile PBS 3 times, cells were lysed with RIPA Lysis Buffer (Beyotime) mixed with phosphatase inhibitor and protease inhibitor (Selleck) or Trizol (Accurate Biotechnology) for further Western Blot.

### 4.6 Hypothalamic stereotaxic injection and cell implantation

Purified AAVs were generated and bilaterally intra‐MBH injected as previously.^10^, ^30^ Adeno‐associated viruses (AAVs) were suspended in 0.5 μl artificial cerebrospinal fluid (aCSF). Mice were anaesthetized with 1% sodium pentobarbital (0.15 ml/20 g) and fixed to the locator. The *X*, *Y*, and *Z* axis values were read using the Bregma point coordinates as the origin. The MBH was located 5.8 mm below the skull surface, 1.7 mm behind the Bregma horizontal line, and 0.25 mm lateral to the midline of the brain.^10^ The cranial surface at the hypothalamic localization was drilled with a miniature handheld cranial drill. The needle was slowly introduced into the hypothalamus, and the micro‐injection pump was fixed to pump the AAVs at a rate of 0.2 μl/min.

The cell implantation method is as previously described.^8^, ^18^ Briefly, cultured htNSCs were labelled with DiI following the instruction of the cell plasma membrane staining kit (Beyotime, China). DiI‐labelled htNSCs were then suspended in 0.5 μl phosphate buffer saline and bilaterally injected into the MBH (9000 cells on each MBH side) using the coordinates. The same injection site as above.

### 4.7 Glucose tolerance test (GTT) and insulin tolerance test (ITT)

The levels of blood glucose were measured by a glucometer monitor (Sinocare). Mice were intraperitoneal injection with glucose (Sigma, 1 g/kg) or insulin (Sigma, 1 U/kg). Blood was taken from the tail vein at 0 min, 15 min, 30 min, 60 min, 90 min, and 120 min to measure blood glucose values respectively.

### 4.8 Blood glucose, serum insulin, and homeostasis model assessment of insulin resistance index (HOMA‐IR)

The levels of blood glucose were measured by a glucometer monitor (Sinocare). The levels of serum insulin were detected by an ELISA kit (Cusabio, China). Homeostasis model assessment of insulin resistance (HOMA‐IR) index was calculated using the following formula: fasting glucose levels (mmol/L) × fasting serum insulin (mIU/L)/22.5.

### 4.9 In vivo insulin signalling assay

Mice fasted for 6 h. Euthanasia was carried out 15 min after the insulin (7 U/kg) injection via a caudal vein. Liver, muscle, and inguinal white adipose tissue (iWAT) were excised and snap frozen for Western Blot detection of insulin signal.

### 4.10 Measurement of blood and liver TC, TG, FFA and serum ALT, AST

Hepatic lipids were extracted with isopropanol in the ratio of liver weight (g):volume (ml) = 1:9. Triglyceride (TG), total cholesterol (TC), free fatty acid (FFA) and alanine transaminase (ALT), aspartate aminotransferase (AST) were measured with a TG kit,

TC kit, FFA kit, ALT kit or AST kit respectively, according to the manufacturers' instructions. All these kits were purchased from Elabscience Company (Wuhan, China).

### 4.11 Western blot analysis

The Western Blot analysis was conducted as previously described.^31^, ^32^ Primary antibodies were rabbit anti‐NF‐κB p65 (1:1000, Cell Signaling, #8242), rabbit anti‐Phospho‐NF‐κB p65 (1:1.000, Cell Signaling, #3033), rabbit anti‐AKT (1:1000, Cell Signaling, #4691), rabbit anti‐Phospho‐AKT (Ser473u) (1:1000, Cell Signaling, #9271), 1:1000, rabbit anti‐GSK3β (1:1000, Cell Signaling, #9315), rabbit anti‐Phospho‐GSK3β(Ser9) (1:1000, Cell Signaling, #9336), rabbit anti‐Insulin Receptor β (IR) (1:1000, Cell Signaling, #3025), rabbit anti‐Phospho‐IRβ (Tyr1135/1136) (1:1000, Cell Signaling, #3024).

### 4.12 Quantitative real‐time PCR (qPCR)

Real‐time PCR was performed as previously described,^33^, ^34^, ^35^ and SYBR Green PCR Master Mix (Accurate Biotechnology, China) was used. The primer pairs used for qRT‐PCR were listed.

**Table:** 

Gene	Forward primer	Reverse primer
Mus *Hnscr*	CTAAGCGAACTCGGGAGC	CACAGCAGGATTGATGGATG
Mus IL1β	CCGTGGACCTTCCAGGATGA	GGGAACGTCACACACCAGCA
Mus IL6	AGTTGCCTTCTTGGGACTGA	TCCACGATTTCCCAGAGAAC
Mus TNFα	CATCTTCTCAAAATTCGAGTGACAA	TGGGAGTAGACAAGGTACAACCC
Homo IL1β	ATGATGGCTTATTACAGTGGCAA	GTCGGAGATTCGTAGCTGGA
Homo IL6	ACTCACCTCTTCAGAACGAATTG	CCATCTTTGGAAGGTTCAGGTTG
Homo TNFα	CCTCTCTCTAATCAGCCCTCTG	GAGGACCTGGGAGTAGATGAG
SCD1	GCGATACACTCTGGTGCTCA	CCCAGGGAAACCAGGATATT
GPAT	CACACGAGCAGGAAAGATGA	GGACTGCATAGATGCTGCAA
PPARγ	CTGGCCTCCCTGATGAATAA	CGCAGGTTTTTGAGGAACTC
ChREBP	CCTCACTTCACTGTGCCTCA	ACAGGGGTTGTTGTCTCTGG
SREBP1c	GGAGCCATGGATTGCACATT	GGAAGTCACTGTCTTGGTTGTTGA
PPARα	AGAGCCCCATCTGTCCTCTC	ACTGGTAGTCTGCAAAACCAAA
CPT1a	ATCGTGGTGGTGGGTGTGATAT	ACGCCACTCACGATGTTCTTC
CD36	TGGTCAAGCCAGCTAGAAA	CCCAGTCTCATTTAGCCAC
FATP1	CAGTGCCACCAACAAGAAGA	CAGCTCGTCCATCACTAGCA
FABP4	AAGGTGAAGAGCATCATAACCCT	TCACGCCTTTCATAACACATTCC

### 4.13 Histological analysis of tissues

Frozen liver sections were stained with Oil Red O. Paraformaldehyde‐fixed and paraffin‐embedded liver sections were examined histologically with hematoxylin and eosin (H&E).

### 4.14 Immunofluorescence assay

The immunofluorescence was conducted as previously described.^36^, ^37^ Primary antibodies were used as follows: anti‐Phospho‐NF‐κB p65 (1:200, Cell Signaling, 3033), anti‐Phospho‐AKT (Ser473u) (1:200, Cell Signaling, 9271), anti‐Sox2 (1:200, Millipore, AB5603), anti‐NeuN (1:500, Millipore, MAB377), anti‐TNFa (1:200, Santa Cruz, sc‐52746).

### 4.15 Statistical analysis

All data are presented as means ± SEM. The statistical significance of the differences between various treatments or groups were evaluated by either Student's *t* test or ANOVA followed by Bonferroni post‐test. Data analyses were performed using GraphPad Prism 7.0. *p* < 0.05 was considered significant.

## AUTHOR CONTRIBUTIONS

Tie‐Jian Jiang designed this study; Ya Liu and Yi‐Fan Guo performed most of the experiments, generated data, and wrote the manuscript; Hui Peng, Hai‐yan Zhou, Tian Su, and Mi Yang analysed the data and designed the figures; Qi Guo, Ye Xiao, Yan Huang, and Tie‐Jian Jiang supervised this study and revised the manuscript.

## CONFLICT OF INTEREST

The authors declare that they have no conflict of interest.

## Supporting information


**Figure S1** *Hnscr* knockout induces liver steatosis. (a, b) Liver weight and appearance. (c) HE and Oil red staining of representative liver sections. Scale Bar: 50 μm. (d–h) Liver and serum TG, TC, FFA level. (i, j) mRNA levels of genes related to lipid metabolism. (k) Serum ALT and AST level. Statistical significance was calculated by two‐tailed Student's *t* test or two‐way ANOVA (*p < 0.05, **p < 0.01, ***p < 0.001).
**Figure S2** Hypothalamic *Hnscr* overexpression did not alter liver steatosis. (a) Liver weight. (b) Liver appearance. (c) HE staining of representative liver sections. Scale Bar: 50 μm.
**Figure S3** Hepatic *Hnscr* overexpression did not alter insulin sensitivity and liver steatosis. AAV‐*Hnscr* and the its control AAV‐Scramble were injected to 2‐month old mice through tail vein, following high‐fat diet‐feed for 3 month. (a) qPCR analysis of *Hnscr* mRNA in liver 1 month after AAVs injection. (b) Fasting and fed blood glucose levels. (c–f) Glucose tolerance tests and insulin tolerance tests. (g, h) Liver weight and appearance. (i–m) Liver and serum TG, TC, FFA level. (n) Serum ALT and AST level. Data are presented as mean ± SEM (*n* = 5–6). Statistical significance was calculated by two‐tailed Student's *t* test (*p < 0.05, **p < 0.01, ***p < 0.001).

## Data Availability

The data that support the findings of this study are available from the corresponding author upon reasonable request.
