# Supplementary material for: Hypothalamic Hnscr regulates glucose balance by mediating central inflammation and insulin signal
Source: Cell Prolif. 2022 Aug 30;56(1):e13332. doi: 10.1111/cpr.13332 (PMC9816933; doi:10.1111/cpr.13332)
Supplement: Supplementary file 1 — Figure S1 Hnscr knockout induces liver steatosis. (a, b) Liver weight and appearance. (c) HE and Oil red staining of representative liver sections. Scale Bar: 50 μm. (d–h) Liver and serum TG, TC, FFA level. (i, j) mRNA levels of genes related to lipid metabolism. (k) Serum ALT and AST level. Statistical significance was calculated by two‐tailed Student's t test or two‐way ANOVA (*p < 0.05, **p < 0.01, ***p < 0.001). Figure S2 Hypothalamic Hnscr overexpression did not alter liver steatosis. (a) Liver weight. (b) Liver appearance. (c) HE staining of representative liver sections. Scale Bar: 50 μm. Figure S3 Hepatic Hnscr overexpression did not alter insulin sensitivity and liver steatosis. AAV‐Hnscr and the its control AAV‐Scramble were injected to 2‐month old mice through tail vein, following high‐fat diet‐feed for 3 month. (a) qPCR analysis of Hnscr mRNA in liver 1 month after AAVs injection. (b) Fasting and fed blood glucose levels. (c–f) Glucose tolerance tests and insulin tolerance tests. (g, h) Liver weight and appearance. (i–m) Liver and serum TG, TC, FFA level. (n) Serum ALT and AST level. Data are presented as mean ± SEM (n = 5–6). Statistical significance was calculated by two‐tailed Student's t test (*p < 0.05, **p < 0.01, ***p < 0.001). [file CPR-56-e13332-s001.docx]

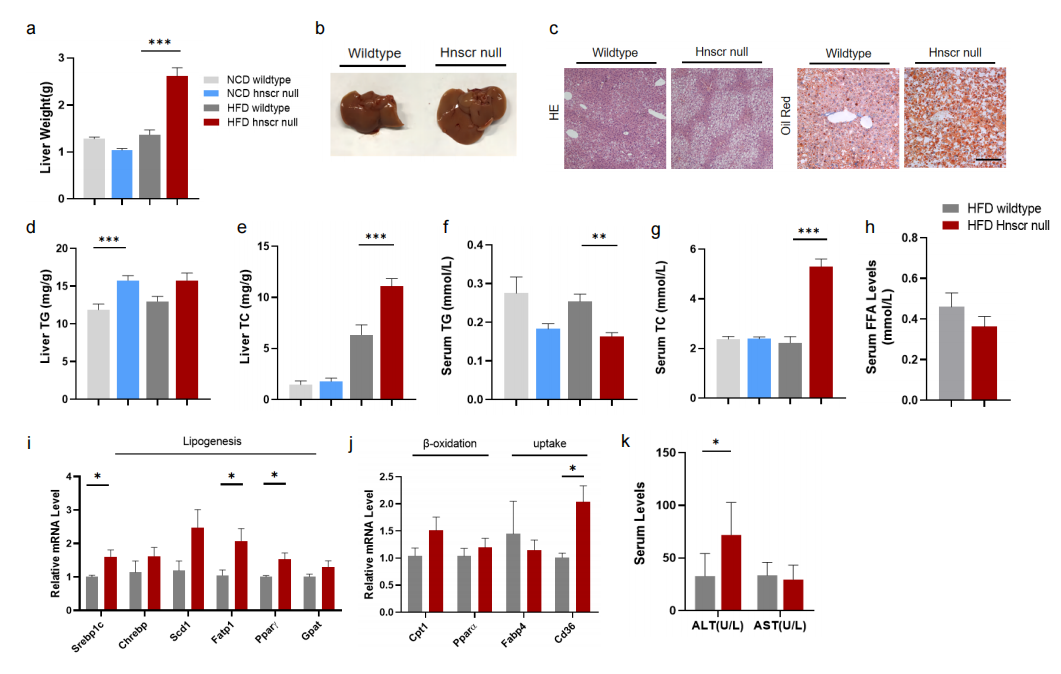


**Supplementary Figure 1 *Hnscr* knockout induces liver steatosis**

(a-b) Liver weight and appearance. (c) HE and Oil red staining of representative liver sections. Scale Bar: 50μm. (d-h) Liver and serum TG,TC, FFA level. (i-j) mRNA levels of genes related to lipid metabolism. (k) Serum ALT and AST level. Statistical significance was calculated by two-tailed Student’s t test or two-way ANOVA (*P<0.05, **P<0.01, ***P<0.001).


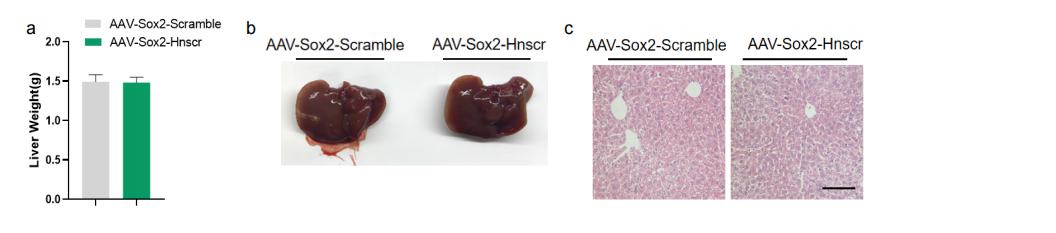


**Supplementary Figure 2 Hypothalamic *Hnscr* overexpression didn’t alter liver steatosis**

(a) Liver weight. (b) Liver appearance. (c) HE staining of representative liver sections. Scale Bar: 50μm.


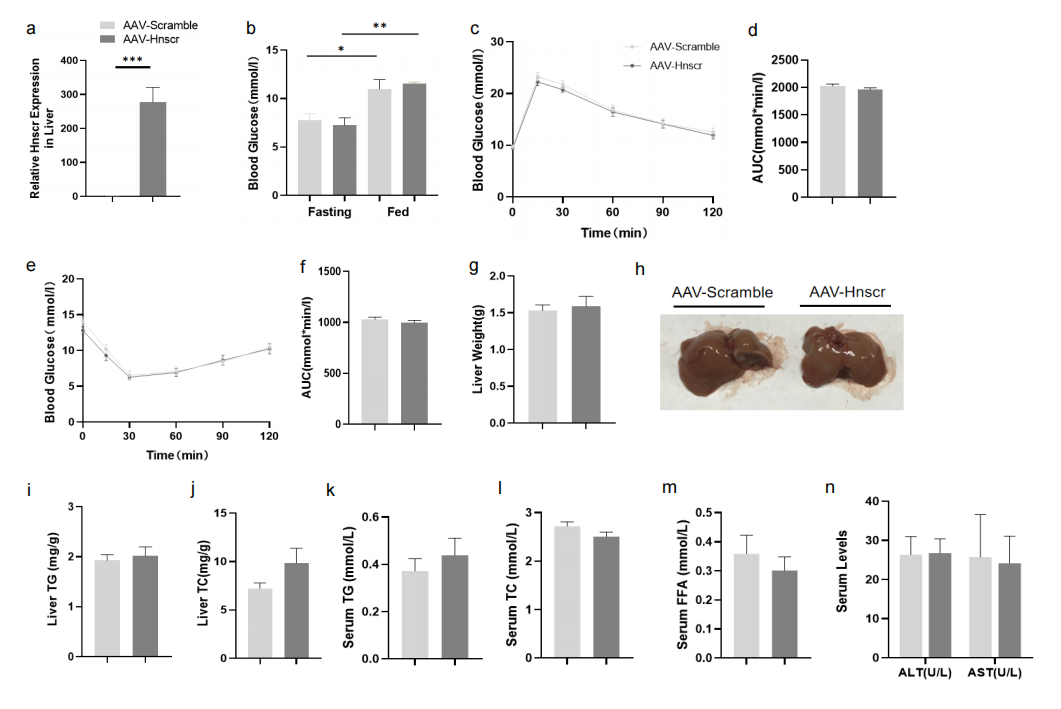


**Supplementary Figure 3 Hepatic *Hnscr* overexpression didn’t alter insulin sensitivity and liver steatosis**

AAV-*Hnscr* and the its control AAV-Scramble were injected to 2-month old mice through tail vein, following high-fat diet-feed for 3 month. (a) qPCR analysis of *Hnscr* mRNA in liver one month after AAVs injection. (b) Fasting and fed blood glucose levels. (c-f) Glucose tolerance tests and insulin tolerance tests. (g-h) Liver weight and appearance. (i-m) Liver and serum TG,TC, FFA level. (n) Serum ALT and AST level. Data are presented as mean ± SEM (n=5-6). Statistical significance was calculated by two-tailed Student’s t test (*P<0.05, **P<0.01, ***P<0.001).
